# Perceived determinants of cardiovascular risk management in primary care: disconnections between patient behaviours, practice organisation and healthcare system

**DOI:** 10.1186/s12875-015-0390-y

**Published:** 2015-12-15

**Authors:** E. Huntink, M. Wensing, M. A. Klomp, J. van Lieshout

**Affiliations:** Radboud University Medical Center, Scientific Institute for Quality of Healthcare (IQ healthcare), PO Box 9101, 6500 HB Nijmegen, The Netherlands

**Keywords:** Primary care, Determinants, Qualitative study, Healthcare professionals, Patients, Cardiovascular disease, Patient’s knowledge

## Abstract

**Background:**

Although conditions for high quality cardiovascular risk management in primary care in the Netherlands are favourable, there still remains a gap between practice guideline recommendations and practice. The aim of the current study was to identify determinants of cardiovascular primary care in the Netherlands.

**Methods:**

We performed a qualitative study, using semi-structured interviews with healthcare professionals and patients with established cardiovascular diseases or at high cardiovascular risk. A framework analysis was used to cluster the determinants into seven domains: 1) guideline factors, 2) individual healthcare professional factors, 3) patient factors, 4) professional interaction, 5) incentives and recourses, 6) mandate, authority and accountability, and 7) social, political and legal factors.

**Results:**

Twelve healthcare professionals and 16 patients were interviewed. Healthcare professionals and patients mentioned a variety of factors concerning all seven domains. Determinants of practice according to the health care professionals were related to communication between healthcare professionals, patients’ lack of knowledge and self-management, time management, market mechanisms in the Dutch healthcare system and motivational interviewing skills of healthcare professionals. Patients mentioned determinants related to their knowledge of risk factors for cardiovascular diseases, medication adherence and self-management as key determinants. A key finding is the mismatch between healthcare professionals’ and patients’ views on patient’s knowledge and self-management.

**Conclusions:**

Perceived determinants of cardiovascular risk management were mainly related to patient behaviors and (but only for health professionals) to the healthcare system. Though health care professionals and patients agree upon the importance of patients’ knowledge and self-management, their judgment of the current state of knowledge and self-management is entirely different.

## Background

In previous decades, mortality due to cardiovascular diseases (CVD) has been substantially reduced, yet CVD remain a major cause of death and suffering in Europe [1]. In the Netherlands, CVD is the leading cause of death for elderly women and second cause of death for elderly men [2]. Multidisciplinary guidelines for cardiovascular risk management (CVRM) provide recommendations for counselling and preventive treatment [3, 4]. The European Society of Cardiology issued a practice guideline; a multidisciplinary working group launched an adaptation in the Netherlands [5]. In many industrialised countries a range of educational programmes and financial incentives have been introduced to enhance the implementation of recommended cardiovascular prevention [6]. Even so, not all eligible patients receive optimal cardiovascular care [7]. Audits in general practices found that 40–60 % of the patients received lifestyle advice [8], 80–90 % received statin and antiplatelet therapy, but 28 % of the practice nurses miscategorised patients at risk for CVD [9] and therefore patients could have underused recommended treatment. In addition, patients’ health-related lifestyle and 10-year risk of CVD mortality does not seem to be improved [10, 11] and treatment targets for blood pressure and cholesterol are not achieved by a great amount of patients [7].

In the Netherlands CVRM is mainly delivered in general practices. In recent years, practice nurses were introduced into almost all general practices in the Netherlands [12, 13]. These practice nurses provide a substantial part of CVRM care, which has been delegated by the general practitioner (GP). Increasing numbers of GPs provide CVRM within the organisation of care groups, which arrange the funding of chronic illness care for typically about 100 GPs. Care groups also monitor performance and provide feedback, using quality indicators that are based on data-extraction from computerized patient records. These care groups facilitate the provision of structured chronic care in general practices based on the principles of the chronic care model [14, 15]. An important element in the chronic care model is well-organized self-management education and support for patients. There is a range of e-health options available for patient education and health promotion, several of which are provided by the Dutch College of General Practitioners [16].

Thus, many conditions seem favourable for high quality CVRM in Dutch primary care. Yet, audits suggest there is still room for improvement. Several years ago studies identified a range of determinants of CVRM in primary care [17–19], but there have been major organizational changes in the general practice since then. More GPs work together in group practices, the practice nurse with CVRM as one of her tasks was introduced more widely, patient-held electronic patient records were introduced and care groups organize CVRM. Also, there is a broad supply of educational and support programs for health professionals concerning CVRM. There is no recent comprehensive research focussing on the determinants of CVRM in the Netherlands. The aim of the current study was to identify determinants of the delivery of CVRM in general practice in the Netherlands based on interviews with health care professionals and patients. We used a previously developed framework with seven domains [20] to categorize the identified factors in a qualitative framework analysis.

## Methods

### Study design

We performed a qualitative study in the Netherlands, using semi-structured interviews that were held between May 2012 and June 2014. The ethical committee of Arnhem and Nijmegen waved approval (nr CD/CMO 1351). The Consolidated criteria for reporting qualitative research (COREQ) [21] and RATS [22] were used for the design and description of this study. This study is part of the Tailored Implementation of Chronic Diseases (TICD) project [23]. The overall aim of the TICD project was to provide insight into methods for tailoring implementation of evidence-based chronic illness care.

### Study population

Participants in this study were healthcare professionals involved in CVRM care and patients with established CVD or at high cardiovascular risk treated in general practices. We used a purposive sampling to ensure diversity of healthcare professionals regarding their professions and considering patients with respect to age, sex and whether they had established CVD or high cardiovascular risk. Patients with established CVD were also invited, because CVRM relates to both primary prevention and to secondary prevention in patients with established CVD to prevent another event. Patients at high cardiovascular risk have a 10 year risk score of 20 % or higher for morbidity and mortality due to CVD based on age, gender, blood pressure level, cholesterol level, smoking status and diabetes mellitus. Healthcare professionals were invited by letter, email or telephone. To recruit patients, four general practitioners and four practice nurses were asked to send written invitations to patients with established CVD or at high cardiovascular risk. Patients who agreed to participate posted their informed consent forms in a postage-paid envelop. The researchers subsequently contacted the participants to make an appointment.

### Data collection

The semi-structured interviews of about 30 min each were divided into three parts (Table 1). The interviews started with a short introduction about the TICD project and information about CVRM. The participants were asked to mention determinants of current practice. During this phase no framework or taxonomy was used and there were no restrictions in number or type of determinants. Next, the interviewer presented the seven domains in the TICD checklist of determinants of practice [20] and then asked the participants if they could think of additional determinants they had not thought of in the first phase. Finally, the interviewer presented the results of previous research. In a previous phase of the TICD project we held group interviews with general practitioners, practice nurses and practice assistants. The plausibly important and changeable determinants mentioned during the group interviews were presented during the interviews. Participants were asked again if they now had suggestions not mentioned before. Healthcare professionals were interviewed in their working environment and patients were interviewed at their homes. After nine interviews with healthcare professionals and eight interviews with patients we performed an interim analysis. During the subsequent interviews with healthcare professionals and patients we introduced the following topics: training of healthcare professionals, feedback for healthcare professionals, budget, target values, role models, and Dutch healthcare policies. All interviews were conducted by three moderators working on the TICD project (E.H. (junior researcher and nurse, university: health science), M.K. (researcher and GP, university: medicine) and a research assistant, vocational training: analyst). The interviewers were familiar with Dutch healthcare and had experience with interviewing, all three used the same interview format to prevent bias. All interviews were audio taped and described verbatim.

**Table 1:** Interview schedule

Parts of the semi structured interviews	Presentation for participants	Question for participants
1^st^ part	1. Introduction of TICD project 2. CVD in the Netherlands 3. CVRM in the general practices 4. Recent research about CVRM in the Netherlands 5. Room for improvement	What factors plays an important role in CVRM care? Why is the current care sub optimal?
2^nd^ part	Seven domains of the TICD framework 1. Guideline factors 2. Individual healthcare professional factors 3. Patient factors 4. Professional interaction facors 5. Incentives and resources 6. Mandate, authority and accountability 7. Social, political and legal factors	Do You have additional determinants?
3^rd^ part	Important and changeable determinants mentioned during the group interviews 1. Awarness and motivation of patients 2. Medical files to support patient care 3. Cooperation between GPs and specialists in hospitals 4. Motivation GPs 5. Better implementation of the ‘care standard’ 6. Financial support 7. Healthy lifestyle supported by the Dutch government	Do You have additional determinants?

### Data analysis

Data-analysis comprised of two phases. In the first phase thematic content analysis was used, which is a qualitative research method focused on describing a phenomenon [24, 25]. The transcribed interviews were analysed using Atlas.ti7 software, started by open coding. All interviews were analysed by one researcher by coding all possible determinants of practice. This researcher also made the codebook. The first three interviews of the healthcare professionals and the first three interviews of the patients were analysed independently by a second researcher to minimise subjectivity and the results were checked for consistency. This second researcher also independently checked the coding of all other interviews. Discrepancies were resolved through discussion. All determinants were described in a clear and concise way so as to be able to compare them, thereby minimising the risk of confusion or misinterpretation.

**Fig. 1:**
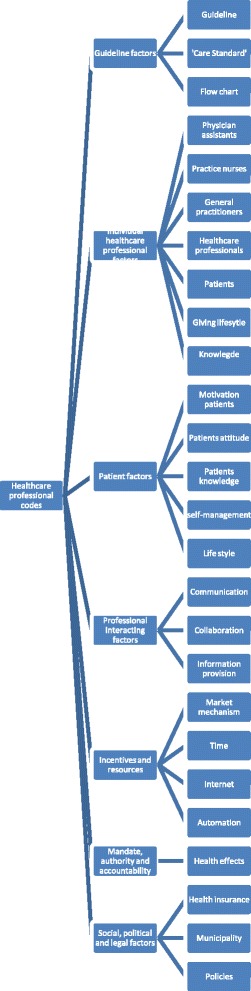
Coding tree for determinants mentioned by healthcare professionals

**Fig. 2:**
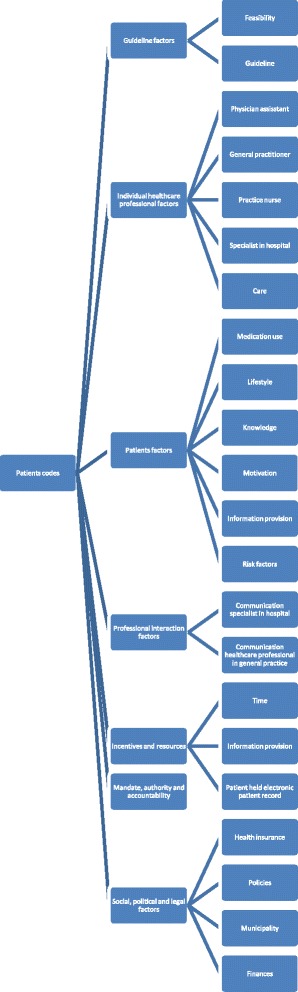
Coding tree for determinants mentioned by patients

For the second phase, whereby axial coding was applied, all determinants were transferred into two Excel data files, one for determinants mentioned by healthcare professionals (Fig. 1) and one file for determinants mentioned by patients (Fig. 2). We used the TICD framework [20] for a framework analysis [26] to cluster the determinants. Determinants were divided into one of the following seven domains: 1) guideline factors, 2) individual healthcare professional factors, 3) patient factors, 4) professional interaction, 5) incentives and recourses, 6) mandate, authority and accountability, and 7) social, political and legal factors. Determinants in each domain were clustered on basis of subthemes. Selective coding was applied by summarising the frequent and important subthemes of the determinants. Axial coding and selective coding were performed by one researcher (E.H.) and checked independently by another researcher (J.v.L). Consensus was reached through discussion.

## Results

### Participants

In total 31 group general practices were approached whereof one practice nurse participated, other healthcare professionals were personally invited and agreed with participation. We have no data on the number of patients approached by the GPs and practice nurses; 16 patients signed the informed consent and were interviewed. The interviews lasted on average 42 min (range 22 to 95 min).

The sample of 12 healthcare professionals consisted of three GPs, an academic GP, a practice nurse and a mental health nurse, a pharmacist, a dietician, a physical therapist, a vascular internist, and an advising GP with a healthcare functionary of a health insurance company (interviewed together). A total of six women and six men participated. Healthcare professionals had a background in 3–6 years of health education from vocational training till university. The sample of participating patients consisted of six women and 10 men, eight patients with established CVD and eight patients at high cardiovascular risk took part (Table 2), they were aged between 59 and 86 years.

**Table 2:** Participans characterisics

Participants		Gender
12 Healthcare professionals	General practitioners (*n* = 3)	Male
	Academic GP (*n* = 1)	Male
	Practice nurse somatic (*n* = 1)	Female
	Practice nurse mental health (*n* = 1)	Female
	Pharmacist (*n* = 1)	Male
	Dietician (*n* = 1)	Female
	Physical therapist (*n* = 1)	Female
	Vascular internist (*n* = 1)	Female
	Advising GP and Healthcare functionary of a health insurance company (*n* = 2)	Male + Female
16 Patients	Patients with established CVD (*n* = 8)	2 Female
		6 men
	Patients at high cardiovascular risk (*n* = 8)	4 Females
		4 men

We will present the results following the TICD framework. First, we will describe the results of the healthcare professionals followed by the results of the patients. Determinants mentioned by healthcare professionals and patients are summarized in Table 3.

**Table 3:** Summary of mentioned determinants by healthcare professionals and patients

	Determinants mentioned by healthcare professionals	Determinants mentioned by patients with established CVD or at high cardiovascular risk
1. Guideline factors	• Practice guideline CVRM • ‘Care Standard’	• Practice guideline CVRM • ‘Care Standard’
2. Individual healthcare professional factors	• Positive about practice nurses • GPs are role models, too busy and clinical inert • Motivational interviewing	• Positive about practice nurses • GPs listen carefully, motivates patients but not always available • Positive about the care and measurements
3. Patient factors	• Not enough knowledge about CVRM • Motivated to improve their health • Not positive about patients self- management • Money can be an obstacle	• Knowledge of a healthy lifestyle • Take good care of themselves • Medication adherence important but difficult due to side effects
4. Professional interaction	• Communication can be improved between GPs and specialists • Collaboration healthcare professionals in general practice is good • Paramedics are important	• Communication between GPs and specialists is rather varied • Collaboration between healthcare professionals in the general practice is good
5. Incentives and recourses	• Time as biggest barrier • Due to ‘open market operation’ more critical look is needed • Digital patient files are helpful but not always accessible	• GP has insufficient time • Information provision is satisfactory • Internet is consulted by half of the patients • Digital patient files are favored
6. Mandate, authority and accountability	• Cannot make health effects provable	
7. Social, political and legal factors	• Dutch government not rated positive • Health insurers should not determine medical policy • GPs responsible for a lot of patients	• The Ministry of Health is much interested in cash excises • Healthcare too expensive • Reimbursements by health insurers • Reforming healthcare

#### 1. Guideline factors

Professionals considered the practice guideline CVRM to be important and clear, but nevertheless expressed that they experienced difficulties in working accordingly. The practice guideline was not seen as easily accessible, feasible, and covering recent insights. The ‘care standard’ with a focus on the organization of CVRM was perceived not to match with current practice; it was not sufficiently matched to specific practice characteristics and was perceived to require a lot of training.

- *I think that those guidelines are currently quite feasible and clear. (healthcare professional (hp) 18)*
- *The guidelines now are too big, too blunt and not liberal enough. (hp 23)*
- *Look, now you have a practice guideline with an endless amount of footnotes. If you want to read it properly then you would need to study all these footnotes, notably because you have to put everything into perspective. I find it a very difficult issue. (healthcare professional (hp) 25)*
- *The care standard is a general guideline and that is fine, but it is by far less differentiated for the general practice, especially for the practice nurse, to effectively work with. (hp26)*

Patients mentioned fewer determinants related to the guideline CVRM or the ‘care standard’. Patients considered the guideline not feasible and thought it did not allow room for own interpretation.

- *The guideline is clear but might create bureaucracy, a stranglehold. Creativity should play a big role. (patient (p) 9)*

#### 2. Individual healthcare professional factors

Healthcare professionals were overwhelmingly positive about practice nurses. Reasons included: practice nurses gave good information and lifestyle advices, formed a role model for patients and created a risk profile for CVRM. Still some critical points were also mentioned; practice nurses did not discuss all CVRM patients with the GP and did not have enough knowledge about mental health problems, which could have impact on life style changes. GPs were seen as role models with a lot of responsibilities; they might have more impact on patients than practice nurses. Important barriers were that GPs sometimes were too busy and clinically inert. Motivational interviewing was perceived to be a promising way of communication with patients. Nevertheless some healthcare professionals said that results of diagnostic tests were not communicated with patients because GPs had no insight into these results or did not check these results. Due to the fact that many patients have co-morbidities, healthcare professionals expressed they have a lack of time for lifestyle counselling.

- *I think that one important thing is, that the professional has no insight into, and does not take the time to check the results of diagnostic tests. (hp 21)*
- *I think that there should be a protocol for CVRM care and a categorical consultation hour just like for the diabetes care, with a practice nurse to guide the consultation because he/she is much brighter than I am (GP). That really works. (hp 24)*
- *Part of the patients has a difficult adjustable hypertension. Sometimes they use four to six different drugs and the systolic blood pressure still is not below 140 mmHg. Sometimes you settle for 160 mmHg. (hp 27)*
- *The practice nurse should pay more attention to the bigger picture; she is now too narrowly focused in her tasks. (hp 28)*

Patients mentioned a lot of positive determinants about practice nurses. Some examples: the consultations went well, the practice nurses gave tailored information, motivated patients, and reserved enough time for consultations. Patients said they had a good relationship with their GP. Positive characteristics of the GP were that they made time available when needed, listened carefully and motivated patients. But on the other hand, patients told that the GP was not always available by telephone, had less patience for the patient and some patients had the feeling that the GP wanted to get rid of them. The practice assistant was considered as positive and friendly but a few patients saw the assistant as an obstacle for visiting the GP. In general, patients were positive about the care they received especially about the frequent measurements. On the other hand, patients experienced a sense of frustration when treatment target values were not achieved: this is disappointing for them, which was not acknowledged by healthcare professionals. Patients needed to be complimented by healthcare professionals and did not want to be ignored. Information provision could be improved; assertive patients received more information which was considered unfair.

- *I have a very good relationship with my GP. He wants to do everything for me, but I cannot contribute to everything. (p 1)*
- *I think that at some point they have to admit that something is nicely done. Just once. (p2)*
- *I had to get used to it, to go to the practice nurse instead of the GP. (p10)*

#### 3. Patient factors

Healthcare professionals’ impression was that patients did not have enough knowledge about CVRM, especially about healthy food. Patients did not always remember given information correctly or understand given information and not all of them were aware that vascular conditions are linked with depressive symptoms. Healthcare professionals found it difficult to explain things about CVRM to patients, in particular the concept of 10-years risk score of 20 % or higher for morbidity and mortality due to CVD is hard to understand for patients. When patients experience no symptoms they find it more difficult to understand why they should prevent CVD or high risk factors. Healthcare professionals consider patients to be motivated to improve their health, but improvement depends on social influences, whereby language and culture issues underlie their motivation. Healthcare professionals did not assess patient’s self-management very positive: patients did not follow lifestyle interventions, forgot appointments, had low therapy compliance, and they stopped prescribed medication. Little interest in CVRM could obstruct changing and managing their lifestyle patterns. Healthcare professionals thought that money could be an obstacle for patients to visit a dietician, purchasing healthcare devices or go to the gym. According to the healthcare professional, only 50 % of the patients do exercise, especially patients with overweigh do not exercise. Impeding factors for not going to exercise were time and a low economic status.

- *Therapy* adherence*, I mean what we face here are also very often language problems, communication problems. (hp 8)*
- *I think that especially in highly educated patients, knowledge about food is overrated. (hp 17)*
- *What we encounter also is that in one way or another, and that continues to be the fact, it is just very difficult to explain something to these people. The conversation with the doctor, well it is still very difficult for some patients to remember things what was said. (hp 18)*
- *Some are aware of it. Not all. Some say: yes my blood pressure was too high. I did not know how high though. (hp 20)*
- *What do you want and what do you need to manage your illness? Well, that is actually the thing we try to promote in our general practice. (hp 26)*

As opposed to the perceptions of healthcare professionals, a large number of participating patients indicated to have sufficient knowledge of a healthy lifestyle, healthy food, their own blood pressure, their health condition, and that they were motivated to take good care of themselves. Patients were aware about the consequences of having a high cardiovascular risk. Due to their healthy lifestyle (less fat, sugar and salt) patients felt much better and that improved their state of mind. Contradictory determinants mentioned by a few: patients were unaware of their health, some were not aware of the importance of a low cholesterol level, thought that lifestyle changes were not feasible and difficult to maintain, and some did not visit their GP for CVRM. Some patients were shocked having a high blood pressure because they were not experiencing any symptoms. Therefore, better education is needed to create more awareness for the patient’s lifestyle and doctors should listen more to patients. Medication adherence was considered to be important, but side effects and changes in medication made therapy adherence difficult. Most patients said to exercise two till seven times a week; especially exercising together was considered as fun and gave them energy. People behaving in a “macho” way at the gym and perceived risk of injuries were some obstacles for exercising.

- *I cannot smoke, I should not eat too much fatty foods, I cannot become overweight, what have I got left? (p 2)*
- *Well yes, what is the difference with other advices, lifestyle advice works differently, it works on my mind. (p 5)*
- *The practice nurse learned me a lot, to eat less salt and eat more regularly. I lost some weight, feel much fitter, eat more regularly and healthier. The practice nurse has guided me well. (p 13)*
- *I exercise a lot and I’m not overweight. (p 15)*
- *Well I think that patients should talk to the doctor and tell him what is going on. Because that is what is going wrong, patients are not assertive enough. (patient 8)*
- *At the pharmacy, they check what they can give you because I also use other medication, and that is just fine. (p 17)*

#### 4. Professional interaction factors

Healthcare professionals stated that communication between GPs and specialists in the hospital could be improved. An example was the difference in which blood pressure or cholesterol levels were accepted. Information subsequently given by the GP or the specialist did not match with each other, resulting in an unclear situation for patients. Some healthcare professionals said that collaboration went well and that GPs got involved in the CVRM care provided by specialists.

Healthcare professionals considered the collaboration between healthcare professionals within general practice as good; they had a clear task differentiation, were aware of each other’s tasks and their level of expertise. Mutual consultations took place on a regular basis, although a few healthcare professionals disagreed on this.

Allied health professionals such as physical therapists and dieticians were also important for the CVRM care. Face-to-face meetings between healthcare professionals seemed important for a workable collaboration and mutual feedback.

- *A lot of explanation about medication use for patients is lacking from the specialist in the hospital, as well as from the GP. A lot of patients think that the prescribed medication is a treatment for two weeks, they do not realize that they have to use this medication for the rest of their lives. (hp 19)*
- *So the face-to-face contact with a GP is very important. A telephone meeting is already better than a letter. When a letter is not read, you do not get connected. (hp 20)*
- *For example, the patient has a broken hip and has been hospitalized. Prior to the operation the cardiologist visits the patient and changes the whole medication schedule without bothering to call the GP. (hp 26)*
- *I ‘am always very clear that I want the systolic blood pressure under the 140, otherwise I ‘am not satisfied. And sometimes patients said that the GP is okay with the blood pressure but I find it to high. (hp 28)*

Patients’ opinions about the communication between GPs and specialists rather varied. An example of good communication was that a specialist sent information such as laboratory results to GPs. Also some patients felt the opposite. Occasionally it happened that a patient wanted to be referred to a hospital-based specialist but the GP did not make the referral. When the patient finally visited the specialist, he/she talked in a negative way about the GP. In a way, the patient then lingers between the GP and the specialist, which was perceived as an uncomfortable position to be in.

Patients indicated that the communication and collaboration between healthcare professionals within the general practice is going well: within the general practice all healthcare professionals gave the same information.

- *The practice nurse consults the GP, and then she tells me what the GP has said. There is a very good collaboration between the GP and practice nurse. (p 4)*
- *There is no collaboration between the specialist and the GP. It could be a lot better. (p 8)*
- *I have the feeling that my GP really tries to keep me from being referred to a specialist as long as possible. (p 14)*

#### 5. Incentives and resources

Healthcare professionals mentioned lack of time as the biggest barrier for the quality of CVRM care. Time prevented them to motivate patients, to give them lifestyle advices or consult other professionals/ colleagues. In particular, GPs suffered from lack of time; they had to do more work in the same timeframe than some years ago and therefore had less time for treatment.

The leaflets in the general practices were considered a good source of information and helpful, giving patients confidence. Internet was perceived as not ideal by healthcare professionals because not every patient could find reliable information on the Internet.

Due to the introduction of market mechanisms in Dutch healthcare, healthcare professionals felt they were more focused on costs. Nowadays reimbursement is partly based on the volume of consultations and procedures. Some healthcare professionals thought these changes were a waste of resources.

Healthcare professionals’ opinion about patient-held electronic patient records was mixed. It was perceived by some as positive because it would enhance patients’ autonomy, improved transparency of data and facilitated information transfer to other healthcare professionals. Some negative aspects were about the ‘integrated care information system’: the system was not easily accessible for GPs, there was no link with hospital systems, and it was perceived as slow, complicated and not stable.

Multidisciplinary care was perceived to be best and most efficiently organized in small organisations, while changes go slow in large organisations.

- *Leaflets and information are good for patient’s confidence and it should give them the feeling that this is about them, the doctor knows me, and not that I am one of those 100,000 patients. That is very important in this district. (hp 19)*
- *The ‘integrated care information system’ is a crappy system; other systems are also not great. Our system is too complicated. The system is not stable, very often it fails and it is slow. (hp 20)*
- *I have been working a long time with cardiovascular risk management. I do not discuss results with patients because it takes ten, fifteen or twenty minutes and then the following patient is waiting for me. I then think I will do it next time and I will then quickly measure the blood pressure and will finished the consultation on time. (hp 24)*
- *Time is the biggest barrier if you really want to educate patients, ask them what they do for exercise, what they eat on average. And it just takes time to motivate the patient to change his/her lifestyle. (hp 27)*

Patients noticed that the GP has insufficient time for CVRM, consultations were going too fast which was perceived as unpleasant. The practice nurse has more time for CVRM patients, which was perceived as positive.

Regarding information provision, the majority of the patients indicated that there were brochures present in the general practice. In one general practice there was a digital screen with information. Half of the patients used Internet to search for information. It was perceived as a reliable source with clear explanations. The other half of the patients had no Internet access or did not use Internet. According to patients there was enough information on the television and in newspapers about CVRM.

Conditions enabling patients to do physical exercise were: a short distance to the gym, getting a trial lesson, a nice group to exercise with, and personal and sympathetic counseling.

Patient-held electronic patient records were in favor by most patients because all data is available in one file. One patient had concerns about the privacy and the CVRM care in case of a computer crash.

Patients indicated that social contacts have been changed over time. It is different these days; neighbors used to know each other. Also, people in the Netherlands are well-fed and enjoy prosperity.

- *One digital file to work in, I have no qualms. (p 8)*
- *Internet is an easy source to find information. (p 10)*
- *If I was someone who visits the general practice every week, then I can imagine that the GP thinks “there he is again”. But the GP has not seen me in a year. And when I visit the GP everything goes very fast and that is not nice. (p 12)*
- *Society has changed quite a bit, as well as social contacts. I greet my neighbors but I do not actually know their names. (p 16)*

#### 6. Mandate, authority and accountability

In this domain only one comment was mentioned by a healthcare professional:

- *Actually, you cannot make health effects provable. (hp 21)*

The patient group did not mention any determinants in this domain.

#### 7. Social, political and legal factors

The healthcare professionals were not positive about general Dutch healthcare policies. A reason for this was the budgetary limitation for health care. The market mechanisms in Dutch healthcare were rated positively; changes were imposed by the government with many negative consequences. Government campaigns and television advertisements about healthy lifestyles were not noticed by half of the Dutch people, as perceived by healthcare professionals but the government continues to promote healthy lifestyles. Healthcare professionals perceived that health insurers were also struggling with money, quality of care and the market mechanisms in Dutch healthcare. They felt that health insurers should not be the ones who determine which treatment or medication is going to be prescribed: it should be about the content instead of the money, healthcare professionals declared. The health insurers were not helpful in the development of the integrated CVRM care. The care for CVRM needs finance, but GPs felt that they get paid less for the care they provide and medical specialists use up a great part of the collective budget. Another negative effect of the budget according to healthcare professionals was that not all patients can be reached and that the drop out of patients in the general practice was due to money. Dutch GPs were responsible for the care of more patients compared to other countries which influenced the CVRM care. There were fewer workplaces for practice nurses and less practice nurses in training will lead to a shortage in the future, thus affecting care also.

- *It should be purely about the content and not the bucks. (hp 17)*
- *I do not think that the government really dares to make any choices. (hp 18)*
- *Healthcare professionals in health centers are more concerned how to perform checkups on the population and thinking about improving quality. There, healthcare professionals receive more specific information about indicators, which stimulates them to think about it. (hp 22)*
- *You cannot rely on health insurances, you will get a contract but you will not know how it goes in the future. Same for the healthcare policies, where do they now take money* from*? (hp 25)*
- *I see health insurers struggle with the market mechanisms in Dutch healthcare to recruit as many costumers they can, but on the other side I see them struggling about money and the quality of care. (hp 26)*

The national healthcare policies were well known by the participating patients. Although the government cannot demand a healthy lifestyle, they should at least promote it. Not all patients saw commercials about healthy lifestyles on the television. Developing a diagnostic center and supporting parents and schools in promoting a healthy lifestyle could be a part of the responsibilities of the municipality.

Health insurers have a big say about the money and Dutch healthcare has become far too expensive: still patients generally receive reimbursement for all their treatments and medication. Therefore a collective health insurance was found important. For some patients it has become a problem to pay for their health insurance or their membership fee for the sports centre. Some patients think that the wages of healthcare professionals are too high, in particular the people in higher echelons. The quality of care would be positively influenced when administrative layers will be removed, because it will lead to a reduction in bureaucracy, said some patients.

- *One of the problems is that I’m not able to pay the contribution for my medication. (p 1)*
- *The health insurance has never put anything in the way, about whatsoever. (p 3)*
- *It is not the lower layer but also the higher echelons, those people want more and more, and they demand more and more. You have to have the money. Salaries are the biggest expense, I think. (p 6)*
- *When administrative layers are gone it will reduce bureaucracy. (p 9)*
- *The municipality is trying to tackle obesity, trying to change the way people within families live. It is not easy to change things. (p 15)*

## Discussion

The determinants of delivering CVRM mentioned by healthcare professionals and patients largely relate to the same domains; there was no systematic difference between the interviews in 2012 and 2014. Both groups mentioned many determinants of CVRM that were related to patient education and patient self-management of health and disease. Furthermore, both felt that the collaboration of healthcare professionals in the general practice was reasonably good, but that the collaboration between healthcare professionals in the general practice and hospital based specialists could be improved. In addition, health care providers had negative feelings about general health policies, the introduction of market mechanisms and a strong role of health insurers in particular, and felt that these posed barriers for improving CVRM. In short, perceived determinants of CVRM were mainly related to patient behaviours and (but only for health professionals) to the healthcare system.

Although there were a lot of similarities, a striking difference was found regarding the perception of patients’ self-management between healthcare professionals and patients. Healthcare professionals held the impression that patients did not have enough knowledge about CVRM and self-management and could need more information. In their opinion patients did not sufficiently adhere to recommended lifestyle, were insufficient adherent to drug therapy and forgot appointments with their healthcare professionals. Determinants such as socio-economic- status, family-related issues and scientific evidence as mentioned in other research were not indicated by healthcare professionals [27, 28]. Healthcare professionals felt that they put a lot of effort in the care for CVRM patients, yet they did not see results in terms of health outcomes. On the other hand, patients perceived that they have sufficient knowledge of CVRM, show sufficient effort to maintain a healthy lifestyle and take medication as prescribed, which are factors that could enhance their quality of life [29]. Other studies suggested, however, that this is not true for all patients [30]. Patients in our study were mostly elderly, so it could be difficult for them to change their lifestyle [31]. Patients mentioned that they were motivated to change their lifestyle, especially by the GP and practice nurse. Patient-centered counseling techniques, such as motivational interviewing, may be applied by healthcare professionals in the general practice [32]. Studies suggest that this is not very effective in patients with diabetes [33] or vascular disease [10] in general practice. A possible explanation is that the counseling technique was not well used, but it is also possible that it was less effective in these patient populations. When a health care professional applies motivational interviewing, patients have to decide what they want to do and the healthcare professional motivates them. Maybe patients are not used to this approach. Also a gap in given information by healthcare professionals may resulted in a reduced self-management of patients [34]. The relationship between healthcare professionals and patients plays also a important role. There were positive but also negative aspects mentioned about this relationship. Healthcare professionals found it their task to inform patients and patients found healthcare opinion important.

Organizational changes have been successfully implemented in general practice [35]. Patients with established CVD or at high cardiovascular risk were listed and invited to regularly visit the GP or practice nurse. Compared to a decade ago, a much higher number of patients eligible for CVRM is reached and receives adequate preventive healthcare. Nevertheless, there is still a challenges to motivate patients to enhance their self-management [18, 36]. Thus, the changes in practice organization are to some extent disconnected from the challenges of counseling patients.

In our search for determinants of CVRM care, several determinants at the level of the health system were mentioned, although they did not seem directly related to healthcare for patients with established CVD or at high cardiovascular risk. Many organizational changes that are favorable for CVRM, such as better reimbursement and improved information technology, are in fact supported by changes in the healthcare system. Nevertheless, healthcare professionals mentioned problems related to recent changes in the healthcare system, which were results of policies of the latest decade. Our study reveals the frustration of healthcare professionals about the market mechanisms introduced by Dutch healthcare policies to enhance the efficiency of healthcare. Due to the market mechanisms health insurers have a big say in drug treatment for instance they make contracts with various suppliers of generic drugs. Changes in the packages of the prescribed medication hold the risk of mistakes in drug intake, additional questions of patients and less medication adherence. GPs are expected to prescribe the cheapest drug. If a more expensive drug has been prescribed, it is possible that the patient does not receive (complete) reimbursement of its costs or GPs face extra administrative tasks.

Perceived determinants of the delivery of CVRM in different domains seemed to have little connection with each other. Patients still struggle with self-management and lifestyle. Individualized self-management support is one way to improve its impact. To empower self-management of patients with established CVD or at high cardiovascular risk new information technologies can be used, such as websites, apps for smart phones, twitter or patient web communities. These technologies are used to tailor support to individual patients’ needs and capabilities, such as presence of depressive symptoms. Patients with established CVD or at high cardiovascular risk are more prone in developing depressive symptoms [37, 38]. For instance, SeMaS is an online tool to support this approach to self-management support, which is currently tested in a cluster randomized trial [39].

This research was pragmatically aimed at developing a tailored intervention. The results reveal that healthcare performed in general practices for patients with established CVD or at high cardiovascular risk is complex. Performance of healthcare professionals in general practices can be approached from several angels for example quality of care or health outcomes measured by patients in general or disease-specific [40]. Healthcare professionals in the general practice are the first point of contact for a wide variety of signs and symptoms, therefore much general knowledge is required. Another angle is knowledge transfer to patients, whereby healthcare professionals should critically think about how they inform patients [41]. Several determinants of practice are not under the influence of healthcare professionals. How active patients are in following a healthy lifestyle is only partly influenced by healthcare professionals [42].

### Strengths and weaknesses

A major strength of the study is that we interviewed both healthcare professionals and patients in our search for determinants of CVRM in primary care. For this study we held 28 interviews in total. Saturation was not planned. The number of interviewed healthcare professionals initially was set at ten; two additional healthcare professionals were interviewed because we were missing two professions that also contribute to CVRM care. On forehand we decided to interview 16 patients based on feasibility within the limits of our research. About the topics ‘individual healthcare professionals factors’, ‘patient factors’ and ‘professional interacting factors’ we were close to saturation in the healthcare professional group as well as in the patients group. In particular about the topics ‘incentives and recourses’, and ‘social, political and legal factors’ a wider variety of determinants was mentioned in both groups. Results showed that healthcare professionals mutually have a different view on these last topics, which was also seen in the patient group. It is possible that we missed important items, especially about these topics mentioned last. We selected various disciplines of healthcare professionals who were involved in CVRM in primary care. The patient group existed of both patients with established CVD and patients at high cardiovascular risk, representing the spectrum of primary and secondary cardiovascular prevention. There was a risk of selection bias regarding the sample of patients. Possibly, patients with high health literacy, who are satisfied with their healthcare, take good care of themselves and get enough exercise were most willing to participate in an interview. Another strength of the study was that we analyzed the findings according the previously developed TICD framework, as this contributes to the accumulation of knowledge.

## Conclusion

Quality of care in general practices is a complex concept, even so for patients with established CVD or at high cardiovascular risk. The complexity of care is experienced at some points different by healthcare professionals in comparison with patients, also a lot of determinants overlapped each other. An important difference was that healthcare professionals think that patients do not have enough knowledge about of risk factors for cardiovascular diseases and self-management and therefore could need more information. Patients think the opposite: they do have knowledge of risk factors for cardiovascular diseases and try to maintain a healthy lifestyle. Healthcare professionals were negative about the healthcare policies of the Netherlands and health insurers; patients on the other hand, were satisfied because there were no problems with reimbursements. Determinants mentioned regarding healthcare professional and patient, organisation and healthcare system were not in connection with each other. Interviews proved to be a productive method to get insight into the views of both health care professionals and patients. We will use these determinants in further research developing an improvement program related to cardiovascular care in general practices.

## Abbreviations

CVD: Cardiovascular disease
CVRM: Cardiovascular risk management
GP: General practitioner
TICD: Tailored Implementation of Chronic Diseases
HP: Healthcare professional
